# Monitoring *Dermacentor reticulatus* Host-Seeking Activity in Natural Conditions

**DOI:** 10.3390/insects11050264

**Published:** 2020-04-25

**Authors:** Zbigniew Zając, Katarzyna Bartosik, Aneta Woźniak

**Affiliations:** Chair and Department of Biology and Parasitology, Medical University of Lublin, Radziwillowska 11 St., 20-080 Lublin, Poland

**Keywords:** *Dermacentor reticulatus*, ticks, host-seeking activity, repetitive tick activity, tick bite exposure

## Abstract

Ticks are hematophagous ectoparasites of humans and animals. These arthropods employ different strategies in their host-seeking activity; most often, it is the “nest”- and “pasture-questing” behaviour. Some species, e.g., *Dermacentor reticulatus*, exhibit both types of activity depending on their developmental stage. The aim of the present study was to investigate the host-seeking activity of adult *D. reticulatus* ticks in the eastern part of Poland. To this end, ticks were collected with the flagging method during their seasonal activity in three different types of habitat. Active specimens were marked with a permanent marker and then released. This was repeated consistently at 7-day intervals using a different colour of the marker each time, which allowed tracking the questing activity of the specimens. Most frequently, repetitive tick activity (repeated up to seven times) was noted in a locality surrounded by urban developments. In an agriculturally unused open meadow habitat, 69.9% of *D. reticulatus* ticks were found to undertake questing activity only once. *D. reticulatus* females proved to be more aggressive and determined to find a host than the males of this species. Adult *D. reticulatus* ticks are able to stay in the habitat for a long time and undertake multiple host-seeking activities. The greatest threat of attacks on animals, including domestic animals, and sporadically humans, by these ticks occurs in meadow habitats, which are preferred by this species.

## 1. Introduction

In the course of evolution, ticks (Acari: Ixodida) have evolved different strategies of searching for a host, whose blood is indispensable for the transition of juvenile forms into the next stage or for initiation of the vitellogenesis process and oviposition in females. Ticks exhibit two dominant types of host-seeking activity: “nest-” and “pasture-questing” behaviour [1].

In the first type, which is characteristic for most argasid ticks, all developmental stages wait, attack, feed, moult, and lay eggs occur in animals’ burrows or nests. Their daily activity is closely synchronised with the activity and presence of their hosts [2]. A characteristic trait of a majority of tick species in this group is the very strong host specificity, i.e., all developmental stages usually ingest blood from the same vertebrate species or even from the same animal [3]. The “nest-questing” behaviour is also observed in some representatives of hard tick species, e.g., *Ixodes lividus* [4].

“Pasture-questing” ixodid ticks seek their hosts in open areas of pastures and meadows and in thickets and forest ecosystems. They climb up vegetation and wait for the host [1]. This activity is mainly determined by prevailing weather conditions, primarily by humidity and air temperature [5,6]. Temperatures close to 0 °C substantially limit the activity of ixodid ticks. For instance, Duffy et al. [7] reported a temperature limit of 4 °C as an apparent threshold of the questing activity of *I. scapularis* ticks. High air temperature values, i.e., above 26 °C, can largely reduce tick host-seeking activity [8]. Loss of water occurring during the long period of waiting for the host, high temperature, or strong winds forces ticks to replenish its content in the body by returning to the level of litter. Therefore, species preferring small-sized mammals and staying closer to the ground level may probably exhibit host-seeking activity for a longer time [1]. The host-seeking behaviour of ticks is also influenced by photoperiods as well as secreted hormones and semiochemical substances (pheromones, allomones, kairomones) [9,10,11,12].

Some tick species, e.g., *Dermacentor reticulatus*, combine the characteristics of both groups described above in their host-seeking strategy, depending on their developmental stage. Larvae and nymphs of this species are characterised by the “nest-questing” behaviour. This is evidenced by the fact that these stages are either not or sporadically collected from vegetation [13]. They usually feed on small rodents [14] or, less frequently, on birds [15] and probably stay permanently in their burrows/nests or in their immediate vicinity. The highest number and activity of immature stages of this species are observed in summer. Larvae most often attack their hosts in June [14]. Engorged larvae moult, thus producing a new generation of nymphs within a month. At this stage, *D. reticulatus* individuals remain active only for one month (July-August): they attack the hosts and transform to adults [16,17].

Adult stages of *D. reticulatus* species exhibit the “pasture-questing” behaviour [1] and most often attack medium- and large-sized mammals [18]. In Central Europe, adults are active throughout the year, with the exception of winter in frosty weather and the presence of snow cover [13,19,20]. In wintertime, ornate cow ticks enter the hormonally regulated diapause stage with minimised metabolic activity. With the return of positive temperatures, they become active again. The evolutionary adaptation to low temperatures gives ticks an advantage in the host-seeking behaviour over other hard tick species, e.g., *I. ricinus* [13]. In ornate cow tick populations, there are two distinct activity peaks. The spring peak is mainly a consequence of the emergence of adults after winter diapause in March or April. The next peak is observed in autumn (September-October), when specimens that have not yet found a host remain active together with the generation that has developed during the year [13]. Depending on the geographical distribution, predominance of one of the peaks is observed in local *D. reticulatus* populations [21,22,23].

*D. reticulatus* adults wait for their hosts on vegetation, mainly on grass or weeds, after climbing to an average height up to 55 cm. They are very sensitive to the smell of potential hosts; hence, they usually gather at the migration routes of large wild mammals, dogs, and humans [16]. In their preferred habitats, the density of ornate cow ticks can be very high, i.e., it can even be 516 specimens/100 m^2^ [24].

The aim of the present study was to investigate the host-seeking behaviour of adult *D. reticulatus* ticks during their seasonal activity in the natural conditions of different types of ecological habitats. The examined tick species is of great epidemiological importance due to its wide geographical distribution range and transmission of many species of pathogenic microorganisms [13,25]. The occurrence of this species is increasingly being recorded in suburbs and urban agglomerations, which raises the risk of infestation of domestic animals and accidental bites of humans [26,27,28,29,30,31].

## 2. Materials and Methods

### 2.1. Study Area

**Figure 1:**
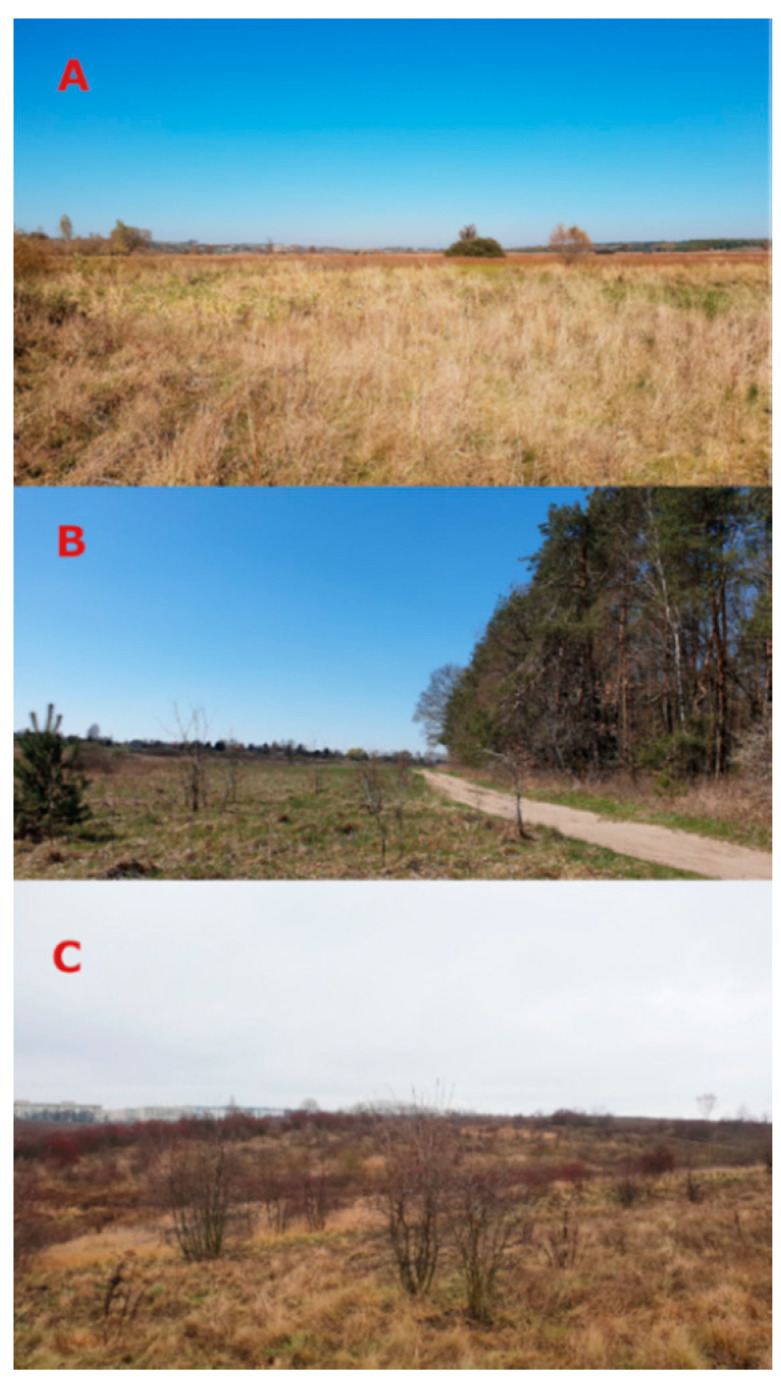
Localities of the research plots. (**A**) plot located in an open meadow, (**B**) plot located in the immediate vicinity of a mixed forest, (**C**) plot located within administrative city limits surrounded by buildings.

The monitoring of the host-seeking activity of *D. reticulatus* ticks in natural conditions was conducted in 2019 in an endemic area of occurrence of this species in eastern Poland (Lublin Province). The investigations were conducted in three habitats differing in their ecological types and designated as localities A, B, and C (Figure 1).

Plot A (51.3540° N, 22.7581° E) was established in an open area on an unused meadow. It was located in close proximity to the Wieprz River and 1.5 km from a large forest complex and was surrounded by arable fields. Plot B (51.3670° N, 22.5473° E) was part of a meadow that was occasionally used as a pasture for cattle. It was situated in the immediate vicinity of a mixed forest and was surrounded by regularly mown meadows and arable fields. Plot C (51.2733° N, 22.5341° E) was established within a city and in close proximity to housing developments. This area is an enclave of natural ecosystems: mainly a meadow ecosystem with patches of synanthropic vegetation and a forest ecosystem formed through progressing ecological succession.

### 2.2. Tick Surveillance

**Figure 2:**
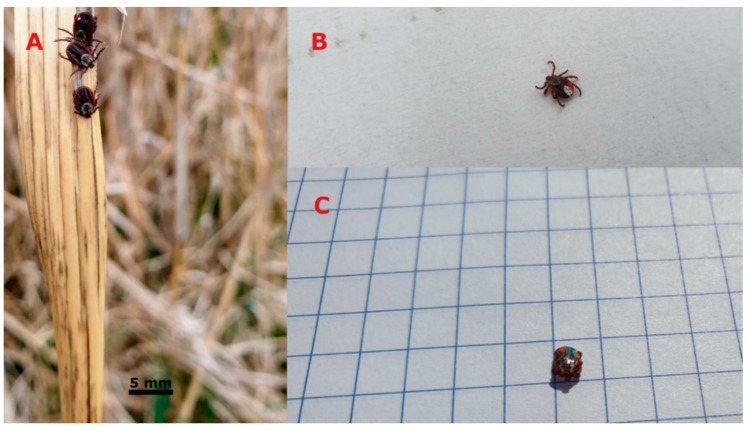
Marked *Dermacentor reticulatus* adult ticks repeating their activity. (**A**) host seeking *D. reticulatus* females; specimen at the top marked with white colour repeating host-seeking activity, (**B**) *D. reticulatus* female active for the third time, (**C**) *D. reticulatus* male active for the fourth time.

The simultaneous field studies were conducted during the seasonal activity of adult *D. reticulatus* ticks in the Lublin region (the study was not conducted in June-August, as adult ornate cow ticks are inactive at this time of the year in eastern Poland) [22,32]. In each site (A with a total area of approx. 6400 m^2^, B approx. 3200 m^2^, C 4000 m^2^), one experimental 100-m^2^ plot was established for collection of ticks on subsequent days. Ticks were collected at equal 7-day intervals with the flagging method. The vegetation in the plots was swept thoroughly with a 1 m^2^ flannel sheet, which was inspected every 2 m. In field conditions, immediately after removal from the sheet, the species and sex of the ticks were identified with the help of a hand magnifier. Specimens identified as *D. reticulatus* based on the morphological features of the species that can be determined in field conditions, i.e., the presence of a *scutum* pattern, the number of festoons, and the structure of palpi [33], were marked with a highly durable permanent oil marker (Figure 2). Next, immediately after marking, they were released at exactly the same place where they were collected. The ticks were marked on the dorsal side of the body: first on the festoons and, if necessary, at the height of the fourth pair of legs. This procedure did not affect their ability to move or their host-seeking activity, as the locomotor and sense organs remained free. Each week, the collected specimens were marked with a different colour, which facilitated monitoring the activity of individual ticks (Figure 2). During each subsequent collection event, previously marked and unmarked individuals were recorded, and collection of marked specimens was regarded as records of the rhythms of repetitive activity.

Each time during the field study, the weather conditions (temperature and relative air humidity) were assessed using the Data Logger R6030 device (Reed Instruments, Wilmington, NC, USA).

### 2.3. Statistical Analysis

Due to the absence of normality of the distribution of the number of active ticks, the differences in the total number of *D. reticulatus* ticks undertaking host-seeking activity in the plots were analysed with the non-parametric Kruskal–Wallis test. The Cochran Q test was used to analyse the distribution of the rhythms of seasonal tick activity (dependent samples) in the individual localities. The statistical analysis of the rhythms of repetitive *D. reticulatus* activity on the next measurement days (dependent samples) was performed using the non-parametric Wilcoxon signed-rank test. The effect of the prevailing weather conditions on tick activity was examined with the Kruskal–Wallis test. The significance level of *p* < 0.05 was adopted in all statistical tests. Statistical analysis was performed using software Statistica 10 PL.

## 3. Results

### 3.1. Seasonal Abundance and Activity of D. reticulatus Ticks

**Figure 3:**
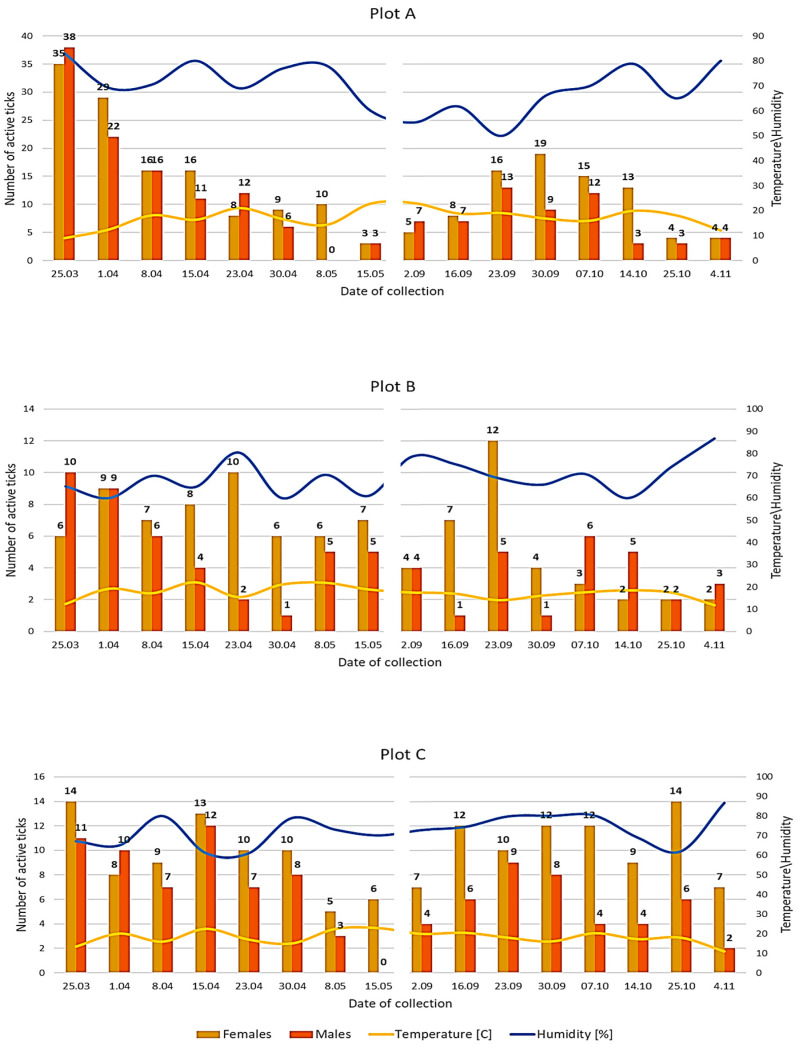
Number of active *Dermacentor reticulatus* adults in context of temperature and relative humidity in the analysed localities in eastern Poland (2019).

Throughout the study period, the highest number of the most active different *D. reticulatus* specimens that exhibited a single or repetitive activity was noted in plot A, i.e., 272 individuals. In plot C, this type of activity was exhibited by 100 ticks, whereas the lowest number of ticks (71) exhibited single or repetitive host-seeking activity in plot B (Table 1). The studied ornate cow tick populations differed significantly in the number of active individuals (H = 10.83, *p* = 0.0044) and the pattern of the rhythms of their seasonal activity (Q = 91.96, *p* = 0.0034). The seasonal activity of *D. reticulatus* adults noted in the particular study plots is presented in Figure 3.

The highest number of active *D. reticulatus* ticks (over 22.0%) was found at the air temperature ranging from 8.9 to 22.5 °C and relative humidity of 60.0–80.0% (Figure 3). The weather conditions prevailing during the field study did not differ significantly between the localities (H = 11.56, *p* = 0.0770) and the activity of ticks was significantly influenced by air temperature (H = 13.5, *p* = 0.0301).

### 3.2. Repetitive Host-Seeking Activity of D. reticulatus Ticks

**Figure A1:**
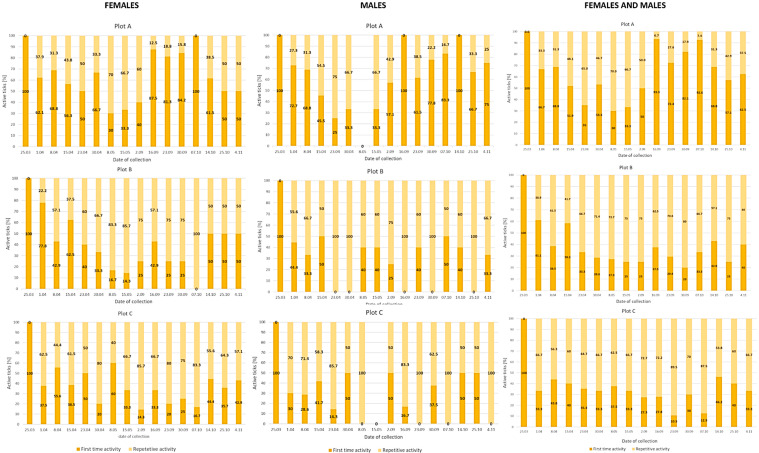
Seasonal distribution of repetitive activity of *Dermacentor reticulatus* adults in particular localities in eastern Poland (2019).

During the 16 measurement days, *D. reticulatus* ticks undertook host-seeking activity from 1 to 7 times (Table 2 and Figure A1). The highest number of specimens that undertook host-seeking activity only once (69.9%) was recorded in the open meadow location, i.e., plot A. In plots B and C, repetitive tick activity was observed more frequently, although the number of active ticks was 3.8-fold (plot B) and 2.7-fold lower (plot C) than in plot A. The questing activity was most frequently repeated twice and three times (35.2% and 18.3%, respectively) by the ticks in plot B. The highest number of ticks that repeated the host-seeking activity four and five times was noted in plot C, i.e., respectively 16.0% and 7.0% vs. 1.8% and 0.4% of ticks in plot A. Six-fold and seven-fold repetition of the host-seeking activity of *D. reticulatus* ticks was observed only in plot C (Table 2).

The number of females vs. males undertaking repetitive activity was statistically significantly higher in each plot (A − T = 16.5, *p* = 0.0427; B − T = 17.5, *p* = 0.0488; C − T = 4.5, *p* = 0.0010). A higher frequency of repetitive activity in the studied tick populations was observed in spring (from March to May) in plot A, whereas no such a relationship was observed in plots B and C throughout the study period (Figure A1).

## 4. Discussion

The present results show that, depending on the ecological habitat type, adult *D. reticulatus* ticks exhibit differences in their host-seeking activity (Table 2 and Figure A1). In the open habitat (plot A) located in the vicinity of the forest, 69.9% of ticks were active once, whereas only 32.0% of ticks undertook only single activity in the urban habitat located in the enclave (plot C) and a majority of specimens exhibited repetitive activity (Table 2). Ecological habitats such as agriculturally unused open meadows are preferred by *D. reticulatus* ticks [13,22,32]. Since they are found in close proximity to forest islands, the habitats provide optimal conditions for the growth of all tick development stages. Given the availability of rodents [34], as well as medium and large mammals [35,36,37], represented in eastern Poland mainly by the elk (*Alces alces*), roe deer (*Capreolus capreolus*), deer (*Cervus elaphus*), and wild boar (*Sus scrofa*) [24], ticks find a potential host more easily than in an urban environment.

The manifold repetitive activity of *D. reticulatus* adults in the site located within the city limits (Table 2) is primarily a consequence of the limited access to hosts. Urban development, even with enclaves of natural vegetation, hinders the migration of large wild-living mammals and is not a place of their frequent visits. In the analysed urban habitat, the host spectrum limitations also affect *D. reticulatus* larvae and nymphs. Studies on the share of rodent species in this area indicate that 87.8% of captured animals were *Apodemus agrarius*, whereas *Microtus arvalis* and *Myodes glareolus*, which are the preferred hosts of juvenile ornate cow ticks, account for only 7.7% [34,38].

Within the ecologically similar types of habitats (plots A and B), we observed clear differences in the abundance and host-seeking activity of *D. reticulatus* adults (Table 2, Figure A1). In our opinion, this is related to the specific location of plot B in the immediate vicinity of regularly mown meadows that are only occasionally used as a pasture for cattle, which limits the presence of ticks in this area [39].

*D. reticulatus* ticks can persist in the environment for a long time. The results of experimental research conducted by Razumowa [23] show that adults are able to survive for 2.5 years (three periods of severe winter in the European part of Russia) maintaining great tolerance to starvation. As suggested by Olsuf’ev [40], adults of this species can survive up to 4 years in an environment with no access to hosts. These observations are confirmed by the results of our study. In each plot, we recorded specimens that showed activity both in the first phase of the study in spring and again undertook host-seeking activity in autumn (Table A1). During our study, we observed a predominant number of active *D. reticulatus* females over males (Figure 3). This is probably related to the fact that females are more resistant to water loss [41] and thus can survive longer in temporarily adverse conditions. Additionally, laboratory studies on the development cycle of *D. reticulatus* have proved that females outnumber males, which may suggest the existence of a genetic mechanism preferring this sex [16]. Moreover, *D. reticulatus* females tend to be more determined (aggressive) in their host-seeking activity (Table 2). The present study showed a statistically significantly higher number of females undertaking repetitive activity. The determination exhibited by female ticks is associated with the necessity to ingest vertebrate blood to lay eggs.

The higher host-seeking activity of *D. reticulatus* ticks during spring (Figure A1, Table A1) facilitates the development of subsequent stages when optimal weather conditions are ensured, which limit the mortality of tick larvae and nymphs exposed to adverse environmental conditions [42,43]. This supports the growth of tick populations in consecutive years and may be the cause of the observed expansion of *D. reticulatus* into new areas [44]. The high abundance of local *D. reticulatus* populations is associated with the risk of infestation of animals, including pets, and sporadic attacks of human. Numerous pathogens have been found in this species in eastern Poland, e.g., *Anaplasma phagocytophilum*, *Rickettsia raoultii*, *Borrelia burgdorferi* s. l., *Babesia* spp., and TBE virus [45].

## 5. Conclusions

Adult *D. reticulatus* ticks are able to stay in the habitat for a long time and undertake repetitive host-seeking activity. The greatest risk of attacks of animals, including pets, and sporadically humans by these ticks occurs in meadow habitats preferred by this species. Due to their increasing occurrence in urban agglomerations and the activity reflected in the incidence of tick-borne pathogen infections in animals and humans, the activity of *D. reticulatus* ticks should be monitored.

## Figures and Tables

**Table 1:** Number of *Dermacentor reticulatus* adults exhibiting activity in the examined localities in eastern Poland (2019).

Plot	Sex	n		Number of Active Ticks				
			Total	Av	SD	Me	Min	Max
A	F	16	152	13.1	±9.0	11.5	3	35
B		16	40	5.9	±3.0	6.0	2	12
C		16	63	9.9	±2.8	10.0	5	14
A	M	16	120	10.4	±9.3	8.0	0	38
B		16	31	4.3	±2.7	4.5	1	10
C		16	37	6.3	±3.3	6.5	0	12
A	F+M	16	272	23.5	±17.7	18.0	6	73
B		16	71	10.3	±4.3	10.0	4	18
C		16	100	16.2	±5.6	17.5	6	25

n—number of measurement days, Av—arithmetic mean, SD—standard deviations, Me—median, Min—minimum value, Max—maximum value.

**Table 2:** Repetitive activity of *Dermacentor reticulatus* adults in the examined plots in eastern Poland (2019).

Repetitions	Plot A						Plot B						Plot C					
	F n = 152		M n = 120		F+M n = 272		F n = 40		M n = 31		F+M n = 71		F n = 63		M n = 37		F+M n = 100	
	n	P [%]	n	P [%]	n	P [%]	n	P [%]	N	P [%]	n	P [%]	n	P [%]	n	P [%]	n	P [%]
1×	108	71.1	82	68.3	190	69.9	10	25.0	11	35.5	21	29.6	24	38.1	8	21.6	32	32.0
2×	34	22.4	33	27.5	67	24.6	16	40.0	9	29.0	25	35.2	9	14.3	14	37.8	23	23.0
3×	6	3.9	3	2.5	9	3.3	7	17.5	6	19.4	13	18.3	14	22.2	3	8.1	17	17.0
4×	4	2.6	1	0.8	5	1.8	3	7.5	3	9.7	6	8.5	9	14.3	7	18.9	16	16.0
5×	0	0.0	1	0.8	1	0.4	4	10.0	2	6.6	6	8.5	4	6.3	3	8.1	7	7.0
6×	0	0.0	0	0.0	0	0.0	0	0.0	0	0.0	0	0.0	3	4.8	1	2.7	4	4.0
7×	0	0.0	0	0.0	0	0.0	0	0.0	0	0.0	0	0.0	0	0.0	1	2.7	1	1.0

F—female, M—male, n—number of specimens, P—percentage proportion of specimens.

## Appendix A

**Table A1:** 10 *Dermacentor reticulatus* adults undertaking the most intensive repetitive activity in the study plots in eastern Poland (2019).

Plot	No. of Tick	Sex	Collection Date															
			25.03	01.04	08.04	15.04	23.04	30.04	08.05	15.05	02.09	16.09	23.09	30.09	07.10	14.10	25.10	04.11
A	1	M	●	●	-	-	●	●	-	-	●	-	-	-	-	-	-	-
	2	F	●	●	●	-	-	-	-	-	●	-	-	-	-	-	-	-
	3	F	●	●	-	-	-	●	-	-	-	-	-	-	-	-	●	-
	4	F	●	●	-	●	-	-	-	-	-	-	-	●	-	-	-	-
	5	F	●	●	-	-	●	●	-	-	-	-	-	-	-	-	-	-
	6	M	-	-	●	●	●	●	-	-	-	-	-	-	-	-	-	-
	7	F	●	●	-	●	-	-	-	-	-	-	-	-	-	-	-	-
	8	F	●	●	-	-	-	●	-	-	-	-	-	-	-	-	-	-
	9	F	●	●	-	-	-	-	●	-	-	-	-	-	-	-	-	-
	10	F	●	●	●	-	-	-	-	-	-	-	-	-	-	-	-	-
B	1	F	●	●	-	-	-	●	-	-	-	●	-	-	-	●	-	-
	2	M	●	-	-	-	-	-	-	●	●	-	-	-	●	●	-	-
	3	F	-	●	-	-	●	-	-	●	●	-	●	-	-	-	-	-
	4	M	-	●	-	●	-	-	●	-	●	-	●	-	-	-	-	-
	5	F	-	-	-	●	●	-	●	●	-	●	-	-	-	-	-	-
	6	M	●	●	●	-	●	-	-	-	-	-	-	-	-	-	-	-
	7	F	-	●	●	-	●	●	-	-	-	-	-	-	-	-	-	-
	8	M	-	●	-	-	-	-	-	-	●	●	-	-	●	-	-	-
	9	F	-	-	-	-	●	●	-	-	-	●	●	-	-	-	-	-
	10	F	●	-	●	-	-	-	-	-	-	-	●	-	-	-	-	-
C	1	M	●	●	●	●	-	●	-	-	●	-	-	-	-	-	-	●
	2	M	-	-	●	●	●	-	-	-	-	●	●	●	-	-	-	-
	3	F	-	-	-	●	●	-	-	-	-	●	●	●	-	-	●	-
	4	F	-	-	-	●	-	-	-	-	-	●	-	●	●	●	●	-
	5	F	-	-	-	-	-	-	●	●	-	-	●	●	-	-	●	●
	6	F	●	●	●	●	-	-	-	●	-	-	-	-	-	-	-	-
	7	F	●	-	●	●	-	-	-	●	-	-	-	-	-	●	-	-
	8	M	●	-	●	-	●	-	-	-	-	●	●	-	-	-	-	-
	9	M	●	-	-	●	-	-	-	-	-	-	-	●	●	-	●	-
	10	M	-	●	-	-	-	-	-	-	●	-	●	●	●	-	-	-

M—males, F—females, ●—activity undertaken by the specimen on the study day, -—no activity undertaken by the specimen on the study day.
